# Human asparagine synthetase associates with the mitotic spindle

**DOI:** 10.1242/bio.038307

**Published:** 2018-11-21

**Authors:** Chalongrat Noree, Elena Monfort, Vorasuk Shotelersuk

**Affiliations:** 1 Institute of Molecular Biosciences, Mahidol University, 25/25 Phuttamonthon 4 Road, Salaya, Phuttamonthon, Nakhon Pathom 73170, Thailand; 2 Section of Cell and Developmental Biology, Division of Biological Sciences, University of California, San Diego, 9500 Gilman Drive (MC 0347), La Jolla, CA 92093-0347, USA; 3 Center of Excellence for Medical Genomics, Department of Pediatrics, Faculty of Medicine, Chulalongkorn University, Bangkok 10330, Thailand

**Keywords:** Asparagine synthetase, Mitotic spindle, Mitosis, Cell division

## Abstract

Cancer cells are characterized by extensive reprogramming of metabolic pathways in order to promote cell division and survival. However, the growth promotion effects of metabolic reprogramming can be due to moonlighting functions of metabolic enzymes as well as the redirection of flux through particular pathways. To identify metabolic enzymes that might have potential moonlighting functions in oncogenesis, we have examined recent screens of the yeast GFP strain collection for metabolic enzymes that have been implicated in cancer metabolism with an unusual subcellular localization. Asparagine synthetase forms filaments in yeast in response to nutrient limitation and is part of a pathway that is a chemotherapy target in acute lymphoblastic leukemia. Interestingly, while yeast asparagine synthetase forms cytoplasmic filaments in response to nutrient stress, human asparagine synthetase is associated with the centrosomes and mitotic spindles. This localization is disrupted by both nocodazole and asparaginase treatments. This failure to localize occurs even though asparagine synthetase is highly upregulated in response to asparaginase treatment. Together, these results argue that human asparagine synthetase undergoes regulated recruitment to the mitotic spindles and that it may have acquired a second role in mitosis similar to other metabolic enzymes that contribute to metabolic reprogramming in cancer cells.

## Abstract (precis)

**Summary:** While yeast Asn1p/ASN2p forms cytoplasmic filaments in response to nutrient limitation, hASNS is associated with centrosomes and mitotic spindles in actively dividing cells, suggesting its additional role in cell division.

## INTRODUCTION

Since the discovery of the Warburg effect in the 1920s, alterations in metabolic activity have long been associated with oncogenesis and tumor progression. However, the last decade has seen an explosion of insights into how these metabolic changes are linked to the rapid growth and development of tumors (DeBerardinis et al., 2008; Hanahan and Weinberg, 2011; Menendez and Alarcón, 2014; Ward and Thompson, 2012). While most of these studies have focused on how various metabolic pathways are optimized to facilitate cell growth and division, recent work has shown that these changes to metabolic enzymes might also drive oncogenesis due to non-metabolic moonlighting functions of the enzyme. One of the best examples to date of this metabolic moonlighting in oncogenesis is the M2 isoform of pyruvate kinase (PKM2). PKM2 alters metabolic activity to provide cancer cells with a growth advantage without the accumulation of ROS (Dong et al., 2016). However, PKM2 can also regulate transcription as either a transcriptional co-activator for HIF-1 or as a protein kinase that targets transcription factors, such as STAT3 (Demaria and Poli, 2012).

Given the possibility that the role of metabolic reprogramming in oncogenesis might rely upon both metabolic and non-metabolic functions of metabolic enzymes, we sought to test whether metabolic enzymes implicated in tumor progression might have acquired moonlighting functions in cell division. For these studies, we focused on asparagine synthetase (ASNS) which synthesizes asparagine from glutamine and aspartate in an ATP-dependent reaction. We focused on ASNS for two reasons. First, asparagine levels are critical for many types of cancer, as shown most dramatically for acute lymphoblastic leukemia (ALL) where asparaginase (ASNase) is used to deplete plasma asparagine levels in chemotherapy. Furthermore, several studies have implicated ASNS as a potential marker for cancer diagnosis in ovarian cancer (Lorenzi et al., 2006), pancreatic cancer (Cui et al., 2007) and prostate cancer (Sircar et al., 2012). Suppression of ASNS has been reported to be able to mitigate cancer and tumor cell growth (Li et al., 2016; Xu et al., 2016; Yang et al., 2014; Yu et al., 2016). Thus, ASNS appears to be a critical enzyme in many types of cancer.

Our second reason for focusing on ASNS is that the yeast asparagine synthetases, Asn1p/Asn2p, are two of the many metabolic enzymes that are capable of polymerizing into novel filaments in response to nutrient limitation (Narayanaswamy et al., 2009; Noree et al., 2010; Petrovska et al., 2014; Shen et al., 2016). While in yeast, the assembly and disassembly of metabolic enzyme filaments is thought to help regulate metabolic pathway activity to help cells survive starvation conditions, it is not known if the ability to form filaments is broadly conserved or if these filaments have acquired additional ‘moonlighting’ functions in higher eukaryotes (Aughey and Liu, 2015; Lynch et al., 2017; Narayanaswamy et al., 2009; Petrovska et al., 2014).

Here we show that while native yeast Asn1p/Asn2p forms cytoplasmic filaments that do not interact with the spindle, human ASNS (hASNS) is recruited to centrosomes and associates with mitotic spindles in actively dividing cells, arguing that the ability of asparagine synthetase to form filaments is evolutionarily conserved. Additionally, we find that ASNase treatment causes both an increase in ASNS expression and a decrease in alpha tubulin expression, while nocodazole treatment only causes a mild decrease in alpha tubulin expression, suggesting that asparagine levels are critical for maintaining the ratio of ASNS to alpha tubulin. Together these results suggest that ASNS may have acquired a moonlighting function in regulating the mitotic spindle.

## RESULTS

### In yeast, asparagine synthetase forms cytoplasmic structures and does not co-localize with microtubules

**Fig. 1.**
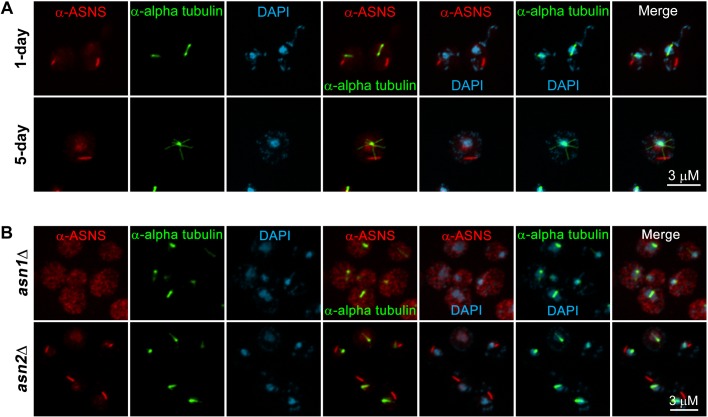
**Co-immunostaining of yeast spheroplasts with anti-hASNS and anti-alpha tubulin revealed that yeast asparagine synthetases formed filaments/foci when grown to saturation and stationary phase.** (A) Yeast BY4741 cells were grown in YPD at 30°C to saturation (1 day) and stationary phase (5 days), respectively. (B) Yeast *asn1Δ* and *asn2Δ* single knockout strains were grown in YPD at 30°C to saturation (1 day). They were all fixed with formaldehyde, lysed their cell walls with zymolase and immunostained with anti-hASNS (red) and anti-alpha tubulin (green), imaged in Z-stack and then compressed into single images using a maximum projection.

Previous screens of the yeast GFP strain collection found that yeast Asn1p and Asn2p can form cytoplasmic foci and filaments in response to nutrient limitation (Narayanaswamy et al., 2009; Shen et al., 2016). However, these studies did not address the possibility that the GFP tag might be responsible for the ability of Asn1p/Asn2p to form filaments. In order to determine if native Asn1p/Asn2p could form filaments as well as develop a reagent that could be used to identify asparagine synthetase structures in other organisms, we generated antibodies against hASNS. Our anti-hASNS was specific for Asn1p and/or Asn2p by immunoblot (Fig. S1). Furthermore, immunostaining with anti-hASNS revealed that native Asn1p/Asn2p formed filaments/foci in yeast grown to either saturation (1-day culture) or the stationary phase (5-day culture) (Fig. 1A; more images shown in Fig. S2). Since yeast has a closed mitosis and these Asn1p/Asn2p structures were present in the cytoplasm, these structures were not spindle associated. Thus, the ability of yeast Asn1p/Asn2p to form filaments is not an artifact of GFP tagging and the structures formed by Asn1p/Asn2p are not associated with the yeast mitotic spindle. Furthermore, single gene deletion analysis suggests that Asn1p is a major contributor to yeast asparagine synthetase assembly and that Asn2p cannot assemble without Asn1p (Fig. 1B).

### Human asparagine synthetase clusters around centrosomes and lines up with mitotic spindles

**Fig. 2.**
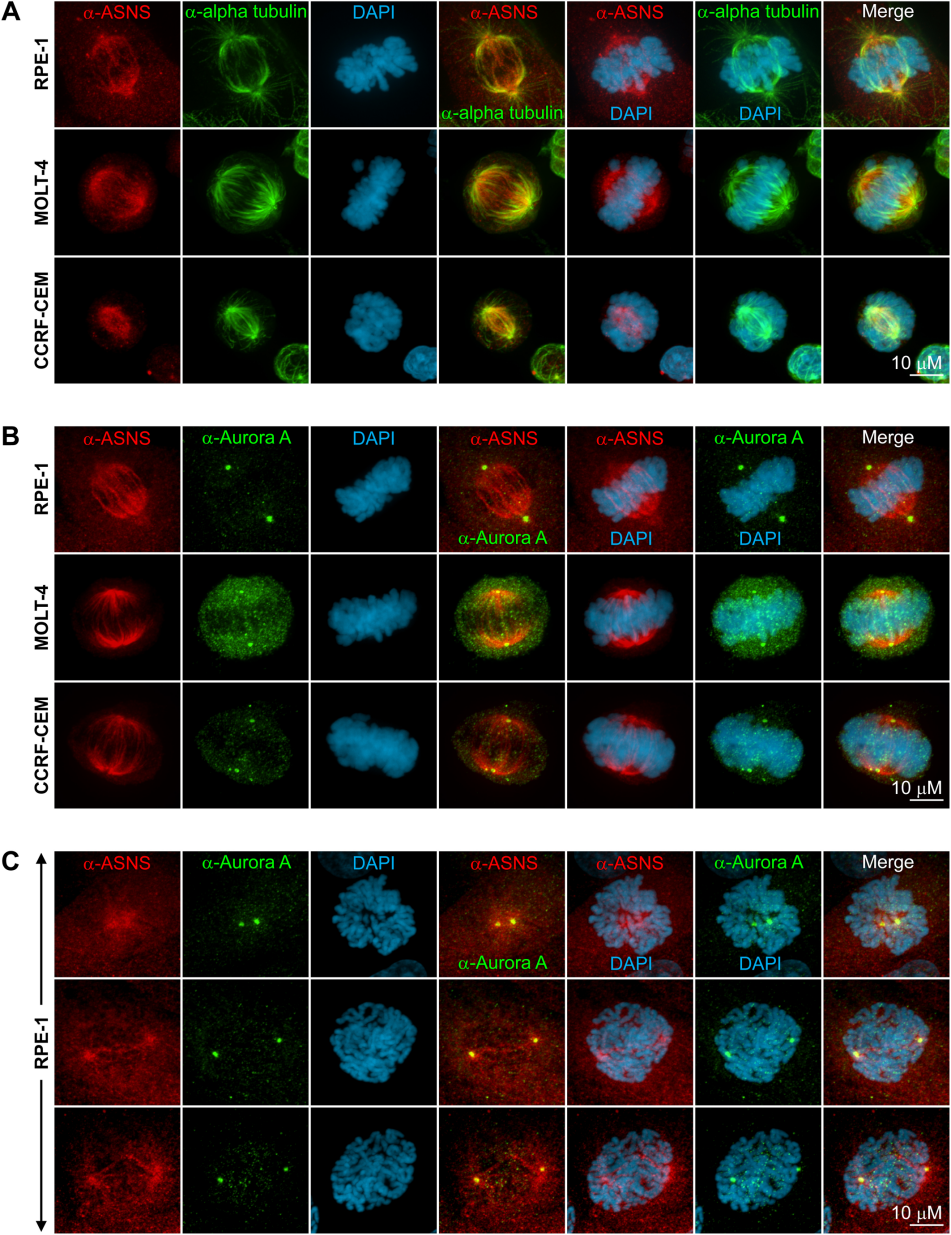
**Co-immunostaining of human cells with anti-hASNS, anti-alpha tubulin and anti-Aurora A revealed ASNS clustering pattern around the centrosomes at the onset of mitosis and mitotic spindle-like structures during mitosis.** RPE-1, MOLT-4 and CCRF-CEM cells were grown for 2 days, fixed with paraformaldehyde and immunostained with anti-hASNS (red) and either anti-alpha tubulin (A) or anti-Aurora A (green) (B,C).

In order to test whether the ability of asparagine synthetase to form structures is evolutionarily conserved, we stained several human cell lines with anti-hASNS and tested the specificity of the anti-hASNS by immunoblot. While our anti-hASNS antibody recognizes a single band by immunoblot, we did not detect any long cytoplasmic ASNS filaments comparable to those we observed in yeast. Instead, we found that hASNS was present in filaments on the mitotic spindle (Fig. 2A,B; more images shown in Figs S3 and S4). Furthermore, asparagine synthetase co-localized with the centrosomal marker Aurora A just prior to the cell's entry into mitosis (Fig. 2C; more images shown in Fig. S5). Not only indirect immunofluorescence, we did also have RPE1 cells stably expressing ASNS-EGFP that showed the mitotic spindle-like pattern (Fig. S6). Together, these results suggest that the ability of asparagine synthetase to form structures has been conserved across evolution, but that these structures may have acquired a novel mitotic function in higher eukaryotes.

### Relative expression of ASNS to alpha-tubulin is increased in cells treated with nocodazole and ASNase

**Fig. 3.**
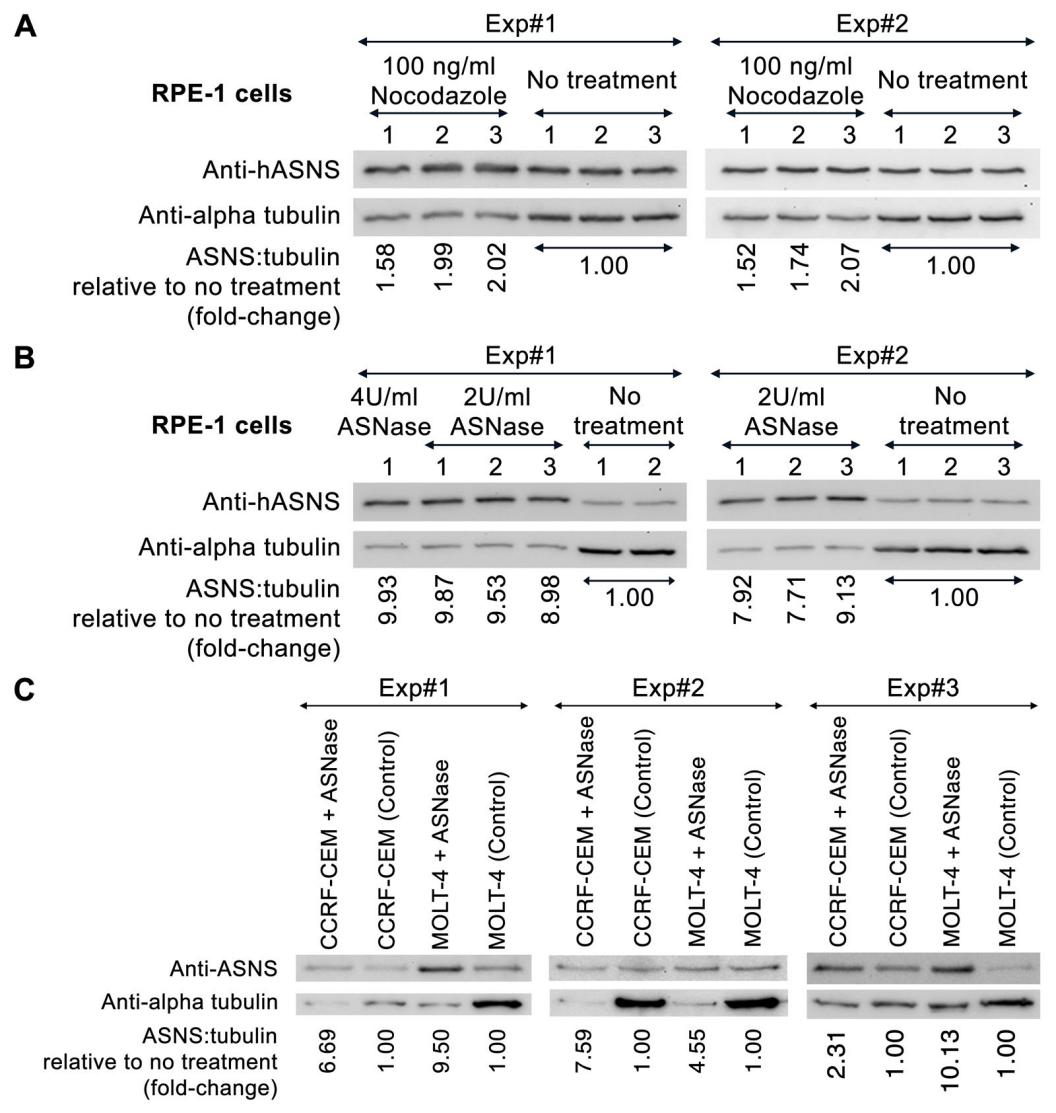
**Relative expression levels of ASNS to α-tubulin were dramatically increased when treating human cells with nocodazole and ASNase.** (A) RPE-1 cells were grown for 2 days, treated with 100 ng/ml nocodazole for 16 h and then harvested for western blot analysis. (B) RPE-1 cells were grown for 2 days, treated with 2 or 4 U/ml ASNase for 3 days and then harvested for western blot analysis. (C) CCRF-CEM and MOLT-4 cells were treated with 2 U/ml ASNase for 2 days and then harvested for western blot analysis. Experiments were independently performed at least twice. ImageJ was used to quantify all western blot data. Full blots are shown in Fig. S7. We study the relative expression ratio between ASNS and α-tubulin, under drug and no drug treatment conditions, that can be expressed as (X_n_/Z_n_)/(Y_n_/Z_n_) which can also be equal to X_n_/Y_n_, therefore loading control (Z_n_) is not required in this case [where X=band intensity of ASNS, Y=band intensity of alpha-tubulin, Z=band intensity of loading control, *n*=sample number]. Fold-change data are then presented.

Given our finding that ASNS was localized to the mitotic spindle, we next explored the effect of different chemotherapy drugs on the expression of ASNS and alpha tubulin. We first tested whether destabilization of microtubules could affect the expression of ASNS by treating RPE-1 cells with nocodazole, which depolymerizes microtubules. Microtubule depolymerization caused a modest increase in relative expression levels of ASNS to alpha-tubulin (∼1.5–2-fold) that was driven largely by a decrease in alpha-tubulin levels (Fig. 3A). In contrast, treatment of RPE-1, CCRF-CEM and MOLT-4 cells with ASNase, an enzyme known to decrease the intracellular asparagine levels, caused both upregulation of ASNS and down regulation of alpha-tubulin. This caused a large change in the relative expression of ASNS to alpha-tubulin ranging from 7.7–9.9-fold in RPE-1 cells, 2.3–7.6-fold in CCRF-CEM cells and 4.5–10.1-fold in MOLT-4 cells (Fig. 3B,C).

**Fig. 4.**
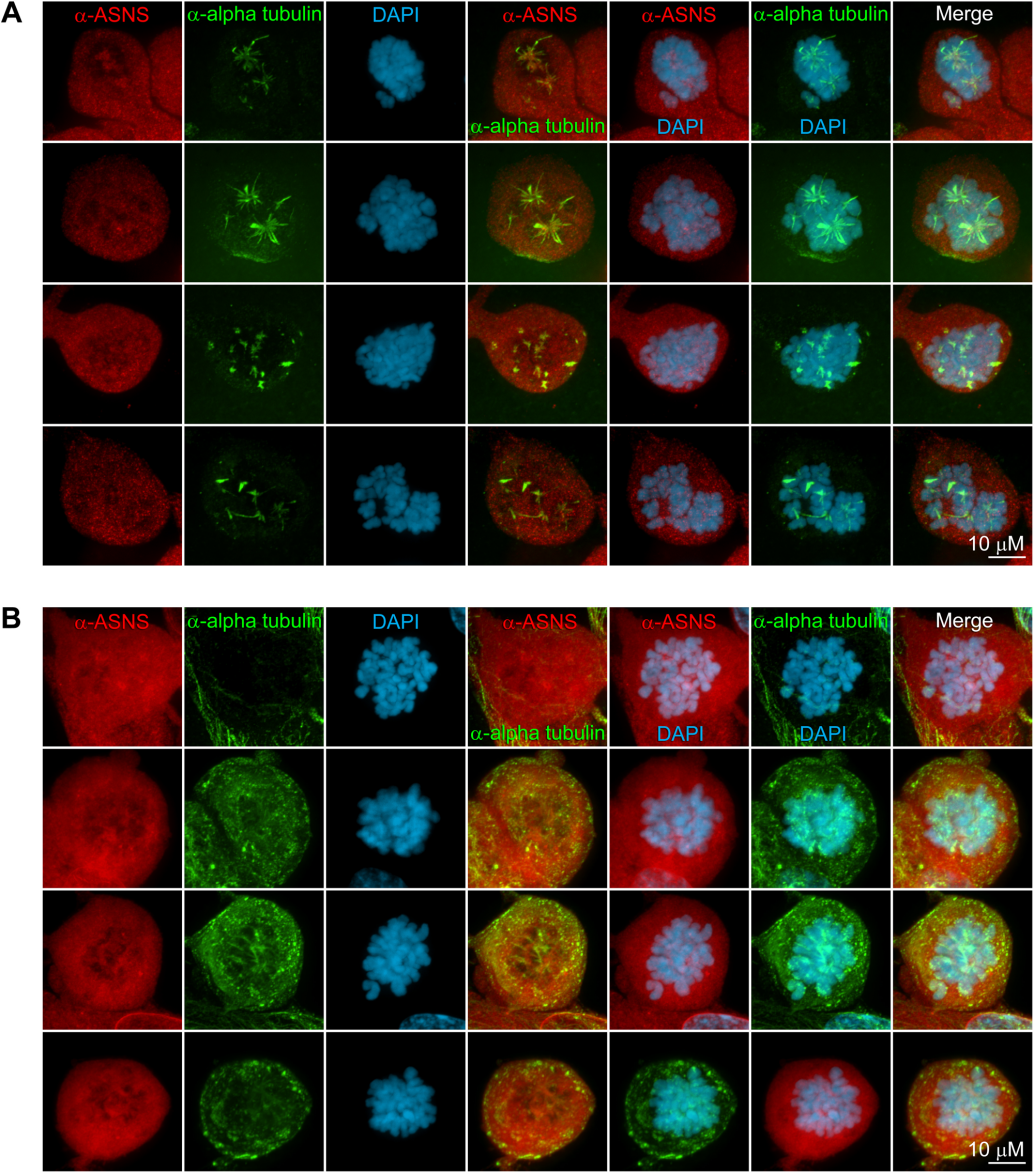
**Co-immunostaining of nocodazole or ASNase treated RPE-1 cells with anti-hASNS and anti-alpha tubulin showed defect in both mitotic spindle formation of tubulins and spindle-like patterning of ASNS.** (A) RPE-1 cells were first grown for 2 days, treated with either 100 ng/ml nocodazole for 16 h (A) or 2 U/ml ASNase for 2 days (B), then fixed with paraformaldehyde, and immunostained with anti-hASNS (red) and anti-alpha tubulin (green).

We next examined the effects of nocodazole or ASNase treatment on the recruitment of ASNS to the spindle. Since nocodazole or ASNase treatment limited the number of actively dividing cells, our analysis was limited to the small fraction of cells that had entered mitosis. Interestingly, both drugs blocked the recruitment of ASNS to the spindle and/or centrosome (Fig. 4A,B) even in those cells where spindle-like structures had formed (Fig. 4A). This suggests that recruitment of ASNS to the spindle is via direct interactions with microtubules and may be regulated by cell cycle checkpoints.

## DISCUSSION

While the number of yeast metabolic enzymes that are capable of forming novel intracellular structures has continued to increase, the assembly behavior of the vast majority of these enzymes has not been explored in other organisms. Here we have confirmed that native yeast asparagine synthetase can form filaments ruling out the possibility that tagging with GFP cause Asn1p/Asn2p to polymerize. Interestingly, while yeast asparagine synthetase forms cytoplasmic filaments that are separate from the mitotic spindle, human asparagine synthetase is recruited to both centrosomes and spindle microtubules. This localization suggests that asparagine synthetase may have acquired a second moonlighting function in mitosis. One possible moonlighting function is that human asparagine synthetase forms structures that associate with the mitotic spindle and play a role in spindle assembly/function. Alternatively, asparagine synthetase might be recruited directly to spindles and centrosomes to provide a localized source of asparagine. While future studies will be required to determine which of these possibilities are correct, they both suggest that chemotherapy with ASNase might have multiple routes to disrupting mitosis than the conventional view that asparagine depletion causes a G1 arrest (Ueno et al., 1997). For instance, our observation that ASNase treatment causes both an upregulation of hASNS and a downregulation of alpha tubulin argues that asparagine levels control both hASNS and alpha tubulin expression. While this effect may be due to a decrease in translation due to loss of asparagine, the localization of hASNS to the spindle raises the possibility that there might be a requirement for high levels of asparagine at the spindle and that this is the critical factor in regulating alpha tubulin expression. Consequently, future work directed at determining how hASNS localizes to the spindle and the role of that localization in regulating spindle assembly and alpha tubulin expression is likely to provide new insights into the anti-cancer effects of ASNase and suggest routes to develop novel anti-mitotic compounds.

## MATERIALS AND METHODS

### Yeast strain and culture medium

Yeast BY4741 (*MATa his3Δ1 leu2Δ0 met15Δ0 ura3Δ0*) and yeast single knockout strains (from the yeast knockout collection) *asn1Δ* and *asn2Δ*; gifts from M. Niwa University of California, San Diego (UCSD), were used in this study, and grown at 30°C to the indicated time point. YPD medium [1% (w/v) yeast extract (BD), 2% (w/v) Bacto-peptone (BD), and 2% (w/v) dextrose (Sigma-Aldrich)] was used for general growth.

### Antibody production

A partial coding region of ASNS (amino acids 251–561) was cloned into pProEx-HTc C and expressed as an N-terminal 6xHis-tagged fusion protein in BL21 (DE3) *Escherichia coli*. His-ASNS was purified under denaturing conditions using a Ni-nitrilotriacetic acid affinity column and injected into rabbits (antiserum production by Covance). Anti-hASNS (CA5498) test bleed#2 was used for all experiments. The specificity of anti-hASNS to yeast Asn1p and Asn2p was tested and shown in Fig. S1.

### Indirect immunofluorescence for yeast cells

Log-phase, 1-day and 5-day cultures of indicated yeast strains were prepared in YPD. Cells were fixed with formaldehyde (1 ml of culture mixed with 100 µl of 37% formaldehyde) for 1 h at room temperature in the dark with shaking. Fixed cells were harvested by centrifugation at 6000 rpm for 3 min. After washing cells twice with 1 ml sterile water, the cells were washed again in 1 ml SK buffer [1 M sorbitol (Sigma-Aldrich), 40 mM K_2_HPO_4_ (Merck) and 7 mM KH_2_PO_4_ (Merck)]. Cells were resuspended and incubated in 0.5 ml SK buffer containing 5 µl beta-mercaptoethanol for 2 min at room temperature. Zymolase (Zymo Research) was then added to lyse yeast cell wall by incubation for at least 20 min at room temperature with gentle shaking. The yeast spheroplasts were harvested by centrifugation at 2000 rpm for 3 min, and after removal of supernatant, they were washed once with 1 ml SK buffer, resuspended in 0.5 ml SK buffer, and then kept on ice until use.

Yeast spheroplasts of indicated yeast strains were put onto a microscopic slide pre-treated with poly-l-lysine (Sigma-Aldrich) and let the cells attach to the slide for at least 10 min (in a humid chamber). After aspirating off supernatant, the slide was soaked in −20°C chilled methanol for 6 min, followed by incubation in −20°C chilled acetone for 30 s. After air-drying for 1–2 min, 1% BSA (in 1×PBS) solution was added to the fixed spheroplasts. The indirect immunofluorescence was performed using standard protocol. Purified rabbit anti-ASNS antibody (CA5498; test bleed#2) (1:500) and Alexa Fluro® 568 goat anti-rabbit IgG (Invitrogen) (1:500) was used to detect yeast Asn1p/Asn2p. Anti-alpha tubulin (12G10, DSHB) (1:1000) and Alexa Fluro® 488 goat anti-mouse IgG (Invitrogen) (1:500) was used to detect yeast microtubules.

### Human cell lines and cell culture

All human cell lines were kindly provided by A. Shiau (Ludwig Institute for Cancer Research, La Jolla). CCRF-CEM and MOLT-4 suspension cells were cultured in RPMI-1640 medium supplemented with 10% FBS at 37°C with 5% CO_2_. RPE-1 adhesion cells were cultured in DMEM/Ham's F12 50/50 supplemented with 10% FBS at 37°C with 5% CO_2_. The cells were counted, passaged and maintained using a standard protocol.

### Indirect immunofluorescence for human cells

5×10^4^ cells were seeded into each well of 6-well plates with two or three sterile coverslips (pre-treated with poly-L-lysine if suspension cells were used). Cells were grown for 2 days, washed once with sterile 1×PBS, fixed with 4% paraformaldehyde (in 1×PBS) for 15 min, followed by another three washes (5-min incubation each) with 1×PBS. The coverslips, having cultured cells attached, were then transferred from the culture plates into a dark moisture chamber for immunostaining. Cells on the coverslips were treated with permeabilization solution (1×PBS, 1% BSA and 0.1% Triton-X100) for 1 h. After aspirating off the permeabilization solution, cells were treated with blocking solution (1% BSA in 1×PBS) for 5 min. The cells were then immunostained. Purified rabbit anti-ASNS antibody (CA5498; test bleed#2) (1:500) and Alexa Fluro® 568 goat anti-rabbit IgG (Invitrogen) (1:500) were used to stain ASNS. Mouse anti-alpha tubulin (12G10, DHSB, University of Iowa) (1:1000) and Alexa Fluro® 488 goat anti-mouse IgG (Invitrogen) (1:500) were used to label the microtubules. Mouse anti-Aurora A (35C1, Invitrogen) (1:333) and Alexa Fluro® 488 goat anti-mouse IgG (Invitrogen) (1:500) were used to label the spindle poles. DAPI (2 µg/ml final concentration) was used to label the nucleus. Immuno-staining with each primary antibody was performed for 1 h at room temperature, or overnight at 4°C. Washing with blocking solution was performed at least three times (5-min incubation each) to remove the unbound primary antibody. After incubation with secondary antibody for 1 h at room temperature, cells on the coverslips were washed four times with 1×PBS (DAPI was applied during the third wash) before mounting the slides with Vectashield (Vector Laboratories).

### Drug treatments and western blot analysis

RPE-1 cells (2×10^5^ cells) were seeded into each well of 6-well plates, and cultured in DMEM/Ham's F-12 50/50 medium supplemented with 10% FBS for 2 days at 37°C with 5% CO_2_. To inhibit microtubule formation, nocodazole (100 ng/ml final concentration) was added to the culture for 16 h. Cells were washed once with sterile 1×PBS prior to protein sample preparation. Cells were scrapped off and collected with RIPA buffer (100 µl/well), and transferred into a microfuge tube. 2×SDS-PAGE sample buffer (100 µl) and sterile glass beads (50 µl) were added to the tube. Samples were vigorously vortexed for 1 min, boiled for 5 min, placed on ice for 5 min, and centrifuged at 10,000 rpm for 1 min. SDS-PAGE and western blotting were performed with a standard protocol (20 µl/sample; 8% SDS-PAGE). ASNS expression levels were detected using rabbit anti-ASNS antibody (CA5498; test bleed#2, purified) (1:500) and HRP conjugated donkey anti-rabbit IgG (1:5000). Tubulin expression levels were detected using mouse anti-alpha tubulin (12G10, DSHB) (1:5000) and HRP conjugated sheep anti-mouse IgG (1:2500). Each experiment used the same blot for probing with anti-hASNS and anti-alpha tubulin, one at a time. Stripping buffer [62.5 mM Tris-HCl pH 6.8, 2% (w/v) SDS, 0.7% (w/v) BME] was used to remove previous antibody from the blot prior to addition of new antibody. Expression levels of ASNS and alpha-tubulin were quantitated with ImageJ (NIH). Full blots are shown in Fig. S7.

To test the effect of ASNase treatment, CCRF-CEM and MOLT-4 were cultured in RPMI-1640 supplemented with 10% FBS with or without 2 U/ml final concentration of ASNase for 2 days. For each treatment, 1×10^6^ cells were collected to prepare the protein sample. Protein sample preparation and western blotting were performed as mentioned above. In case of RPE-1 cells, 2×10^5^ cells were seeded into each well of 6-well plate, cultured in DMEM/Ham's F-12 50/50 medium containing 10% FBS, supplemented with or without 2 U/ml (final concentration) of ASNase for 3 days. The RPE-1 cells were not counted, but instead used from the whole well for sample preparation as the expression level of ASNS was normalized by that of alpha-tubulin before the comparison of the ASNase effect between treated and non-treated conditions was made.

### Confocal imaging

Images were acquired using spinning disk Carl Zeiss Axiovert 200 M microscope with a Plan-Apochromat 100X/1.40 Oil objective lens, and Micro-Manager operation software version 1.4.17. Z-stack images of each sample were taken every 0.25 microns over 3–10 microns, deconvolved and then compressed into a single image.

## Supplementary Material

Supplementary information

## References

[BIO038307C1] Aughey G. N. and Liu J.-L. (2015). Metabolic regulation via enzyme filamentation. *Crit. Rev. Biochem. Mol. Biol.* 51, 282-293. 10.3109/10409238.2016.1172555 27098510 PMC4915340

[BIO038307C2] Cui H., Darmanin S., Natsuisaka M., Kondo T., Asaka M., Shindoh M., Higashino F., Hamuro J., Okada F., Kobayashi M. et al. (2007). Enhanced expression of asparagine synthetase under glucose-deprived conditions protects pancreatic cancer cells from apoptosis induced by glucose deprivation and cisplatin. *Cancer Res.* 67, 3345-3355. 10.1158/0008-5472.CAN-06-2519 17409444

[BIO038307C3] DeBerardinis R. J., Lum J. J., Hatzivassiliou G. and Thompson C. B. (2008). The biology of cancer: metabolic reprogramming fuels cell growth and proliferation. *Cell Metab.* 7, 11-20. 10.1016/j.cmet.2007.10.002 18177721

[BIO038307C4] Demaria M. and Poli V. (2012). PKM2, STAT3 and HIF-1alpha: the Warburg's vicious circle. *JAKSTAT* 1, 194-196. 10.4161/jkst.20662 24058770 PMC3670244

[BIO038307C5] Dong G., Mao Q., Xia W., Xu Y., Wang J., Xu L. and Jiang F. (2016). PKM2 and cancer: The function of PKM2 beyond glycolysis. *Oncol. Lett.* 11, 1980-1986. 10.3892/ol.2016.4168 26998110 PMC4774429

[BIO038307C6] Hanahan D. and Weinberg R. A. (2011). Hallmarks of cancer: the next generation. *Cell* 144, 646-674. 10.1016/j.cell.2011.02.013 21376230

[BIO038307C7] Li H., Zhou F., Du W., Dou J., Xu Y., Gao W., Chen G., Zuo X., Sun L., Zhang X. et al. (2016). Knockdown of asparagine synthetase by RNAi suppresses cell growth in human melanoma cells and epidermoid carcinoma cells. *Biotechnol. Appl. Biochem.* 63, 328-333. 10.1002/bab.1383 25858017

[BIO038307C8] Lorenzi P. L., Reinhold W. C., Rudelius M., Gunsior M., Shankavaram U., Bussey K. J., Scherf U., Eichler G. S., Martin S. E., Chin K. et al. (2006). Asparagine synthetase as a causal, predictive biomarker for L-asparaginase activity in ovarian cancer cells. *Mol. Cancer Ther.* 5, 2613-2623. 10.1158/1535-7163.MCT-06-0447 17088436

[BIO038307C9] Lynch E. M., Hicks D. R., Shepherd M., Endrizzi J. A., Maker A., Hansen J. M., Barry R. M., Gitai Z., Baldwin E. P. and Kollman J. M. (2017). Human CTP synthase filament structure reveals the active enzyme conformation. *Nat. Struct. Mol. Biol.* 24, 507-514. 10.1038/nsmb.3407 28459447 PMC5472220

[BIO038307C10] Menendez J. A. and Alarcón T. (2014). Metabostemness: a new cancer hallmark. *Front. Oncol.* 4, 262 10.3389/fonc.2014.00262 25325014 PMC4179679

[BIO038307C11] Narayanaswamy R., Levy M., Tsechansky M., Stovall G. M., O'Connell J. D., Mirrielees J., Ellington A. D. and Marcotte E. M. (2009). Widespread reorganization of metabolic enzymes into reversible assemblies upon nutrient starvation. *Proc. Natl. Acad. Sci. USA* 106, 10147-10152. 10.1073/pnas.0812771106 19502427 PMC2691686

[BIO038307C12] Noree C., Sato B. K., Broyer R. M. and Wilhelm J. E. (2010). Identification of novel filament-forming proteins in Saccharomyces cerevisiae and Drosophila melanogaster. *J. Cell Biol.* 190, 541-551. 10.1083/jcb.201003001 20713603 PMC2928026

[BIO038307C13] Petrovska I., Nuske E., Munder M. C., Kulasegaran G., Malinovska L., Kroschwald S., Richter D., Fahmy K., Gibson K., Verbavatz J. M. et al. (2014). Filament formation by metabolic enzymes is a specific adaptation to an advanced state of cellular starvation. *Elife*, **3**, e02409. 10.7554/eLife.02409 PMC4011332 24771766

[BIO038307C14] Shen Q.-J., Kassim H., Huang Y., Li H., Zhang J., Li G., Wang P.-Y., Yan J., Ye F. and Liu J.-L. (2016). Filamentation of metabolic enzymes in saccharomyces cerevisiae. *J. Genet. Genomics* 43, 393-404. 10.1016/j.jgg.2016.03.008 27312010 PMC4920916

[BIO038307C15] Sircar K., Huang H., Hu L., Cogdell D., Dhillon J., Tzelepi V., Efstathiou E., Koumakpayi I. H., Saad F., Luo D. et al. (2012). Integrative molecular profiling reveals asparagine synthetase is a target in castration-resistant prostate cancer. *Am. J. Pathol.* 180, 895-903. 10.1016/j.ajpath.2011.11.030 22245216 PMC4715215

[BIO038307C16] Ueno T., Ohtawa K., Mitsui K., Kodera Y., Hiroto M., Matsushima A., Inada Y. and Nishimura H. (1997). Cell cycle arrest and apoptosis of leukemia cells induced by L-asparaginase. *Leukemia* 11, 1858-1861. 10.1038/sj.leu.2400834 9369418

[BIO038307C17] Ward P. S. and Thompson C. B. (2012). Metabolic reprogramming: a cancer hallmark even warburg did not anticipate. *Cancer Cell* 21, 297-308. 10.1016/j.ccr.2012.02.014 22439925 PMC3311998

[BIO038307C18] Xu Y., Lv F., Zhu X., Wu Y. and Shen X. (2016). Loss of asparagine synthetase suppresses the growth of human lung cancer cells by arresting cell cycle at G0/G1 phase. *Cancer Gene Ther.* 23, 287-294. 10.1038/cgt.2016.28 27444726

[BIO038307C19] Yang H., He X., Zheng Y., Feng W., Xia X., Yu X. and Lin Z. (2014). Down-regulation of asparagine synthetase induces cell cycle arrest and inhibits cell proliferation of breast cancer. *Chem. Biol. Drug Des.* 84, 578-584. 10.1111/cbdd.12348 24775638

[BIO038307C20] Yu Q., Wang X., Wang L., Zheng J., Wang J. and Wang B. (2016). Knockdown of asparagine synthetase (ASNS) suppresses cell proliferation and inhibits tumor growth in gastric cancer cells. *Scand. J. Gastroenterol.* 51, 1220-1226. 10.1080/00365521.2016.1190399 27251594

