# Supplementary material for: Human asparagine synthetase associates with the mitotic spindle
Source: Biol Open. 2018 Nov 21;7(12):bio038307. doi: 10.1242/bio.038307 (PMC6310878; doi:10.1242/bio.038307)
Supplement: Supplementary information [file biolopen-7-038307-s1.pdf]

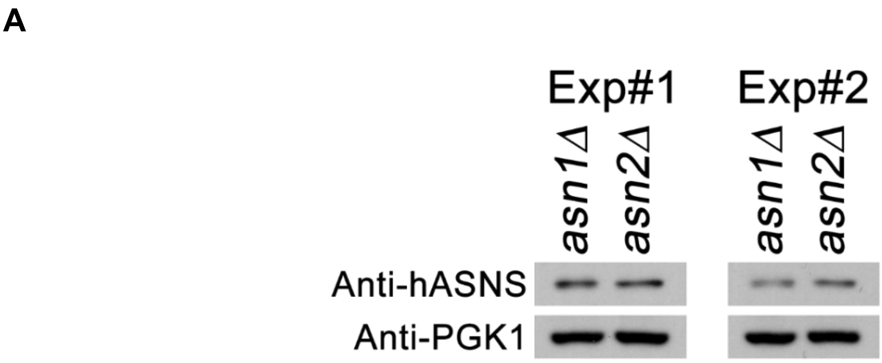

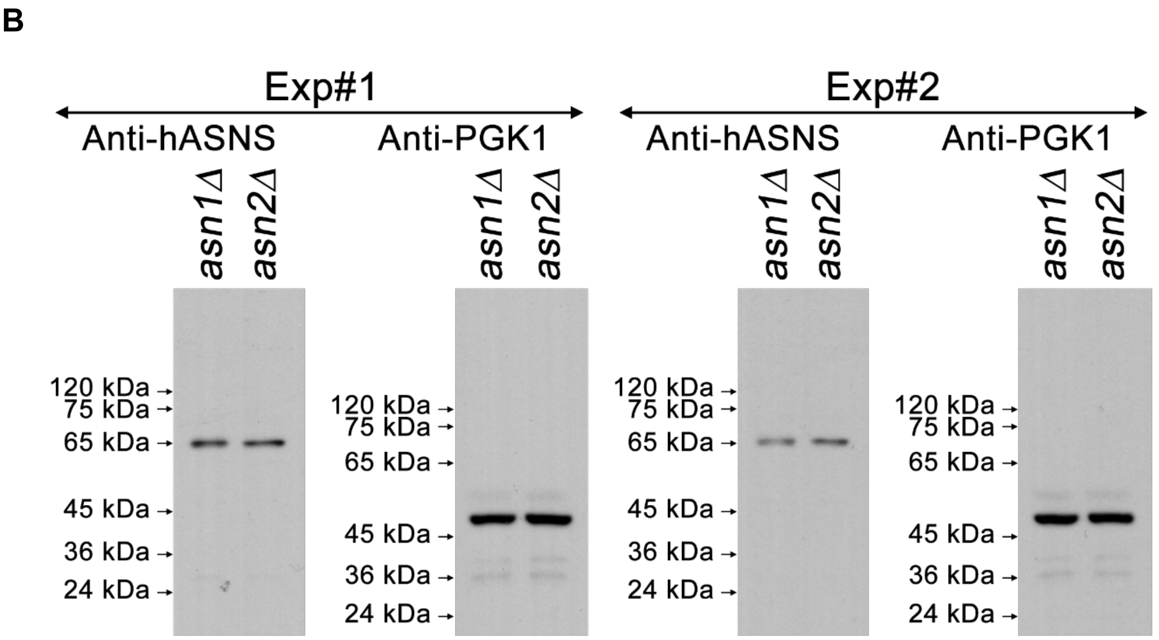

Fig. S1. Western blot analysis of yeast *asn1*Δ or *asn2*Δ strains with anti-hASNS. Indicated yeast strains were grown in YPD for 1 day at 30°C with shaking. Five OD cells were collected for preparing 200-μl whole cell extracts. Ten μl of each protein sample were resolved on 8% acrylamide gel. AccuProtein Chroma prestained protein marker (Enzmart Biotech) was used as size standards. Anti-hASNS detected a single protein band of about 65 kDa (Molecular weight predicted for Asn1p is 64.46 kDa, and 64.57 kDa for Asn2p). Anti-PGK1 monoclonal antibody (22C5D8, 459250) was used to detect yeast 3-phosphoglycerate kinase (44.74 kDa) as an internal loading control. Cropped blots are shown in (**A**), and full blots are present in (**B**). The *asn1Δ* and *asn2Δ* double knockout is inviable, therefore cannot be included for analysis here. However, the Asn1p-GFP expressed by yeast *ASN1::GFP* can be detected by this anti-hASNS (C. Noree, unpublished data).

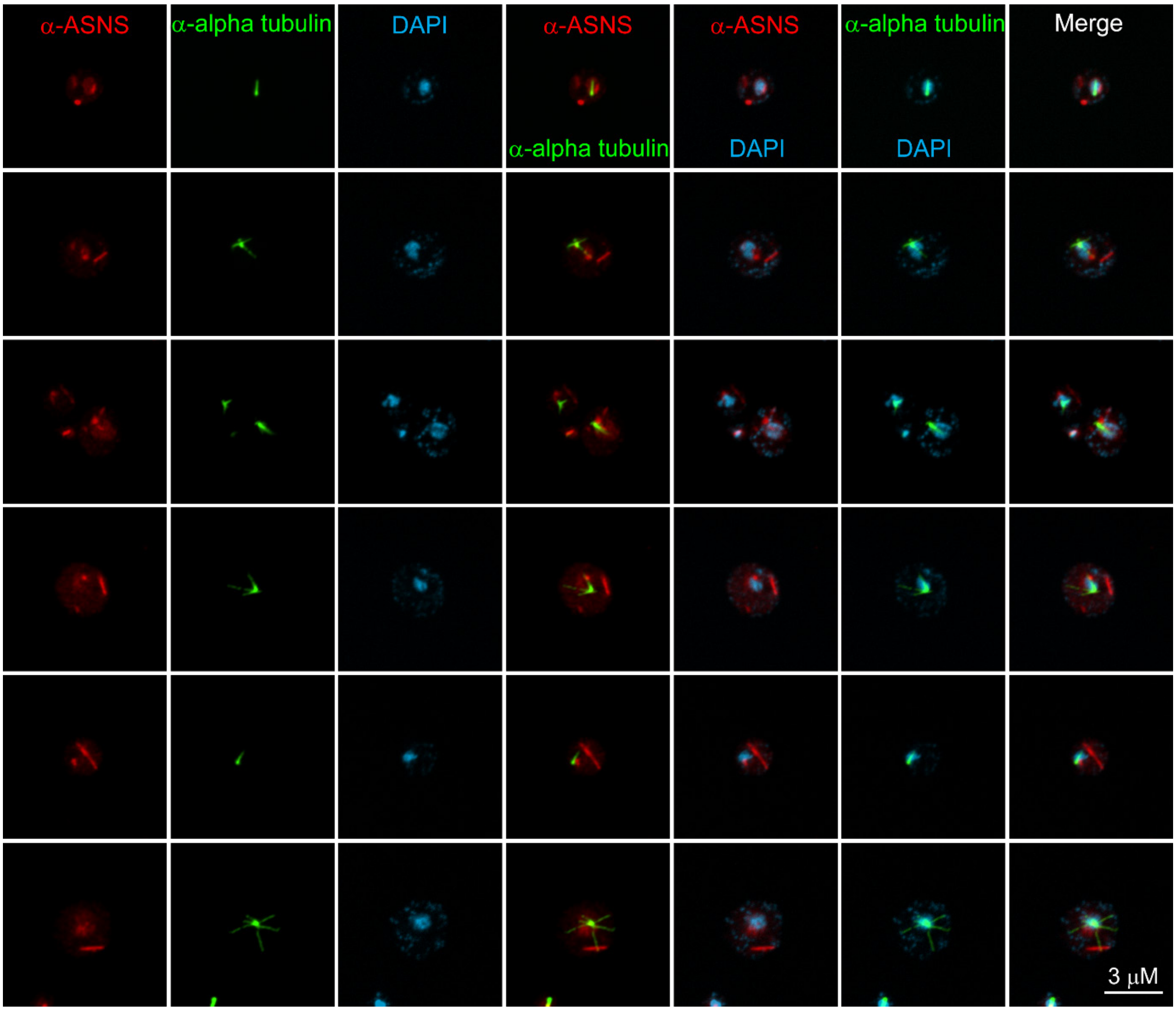

Fig. S2. At stationary phase (5-day culture), yeast asparagine synthetase assembled into cytoplasmic filaments. Yeast BY4741 cells were grown in YPD at 30°C for 5 days, fixed with formaldehyde, lysed their cell walls with zymolase, and immuno-stained with anti-hASNS (red) and anti-alpha tubulin (green).

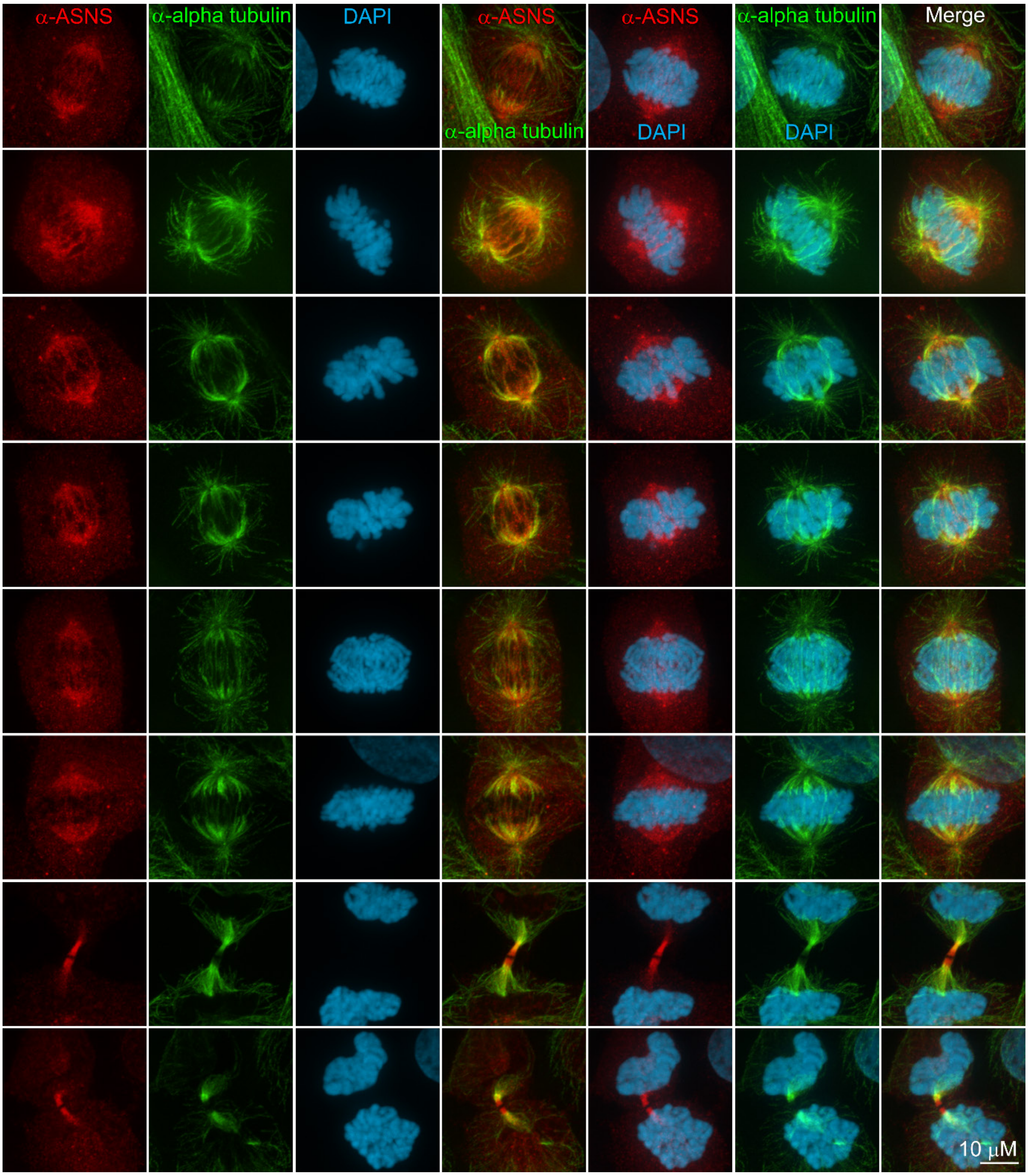

Fig. S3. Human asparagine synthetase lined up with mitotic spindles during mitosis. RPE-1 cells were grown for 2 days, fixed with paraformaldehyde, and immuno-stained with anti-hASNS (red) and anti-alpha tubulin (green).

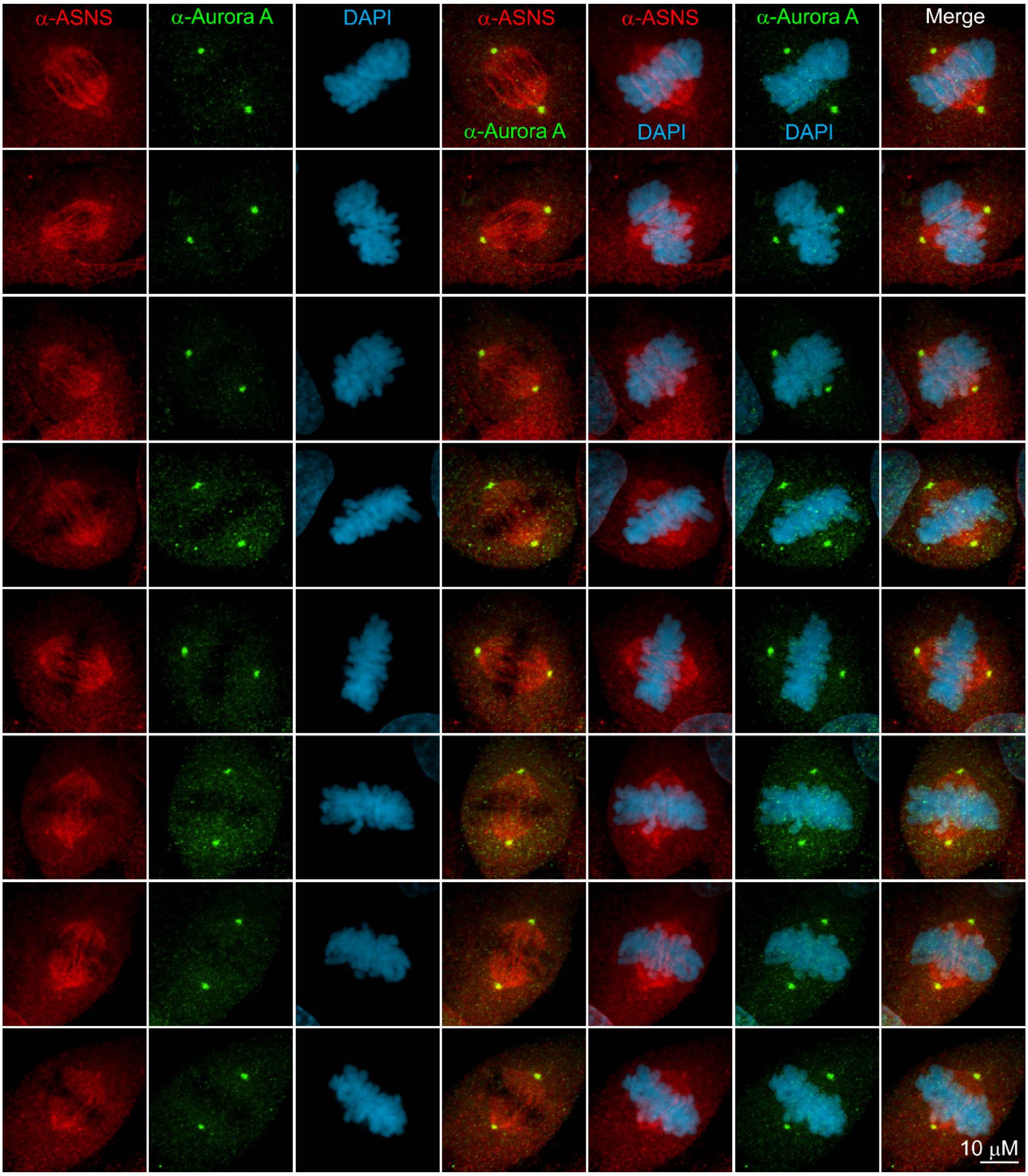

Fig. S4. Human asparagine synthetase showed mitotic spindle-like pattern during mitosis. RPE-1 cells were grown for 2 days, fixed with paraformaldehyde, and immuno-stained with anti-hASNS (red) and anti-Aurora A (green).

Biology Open • Supplementary information

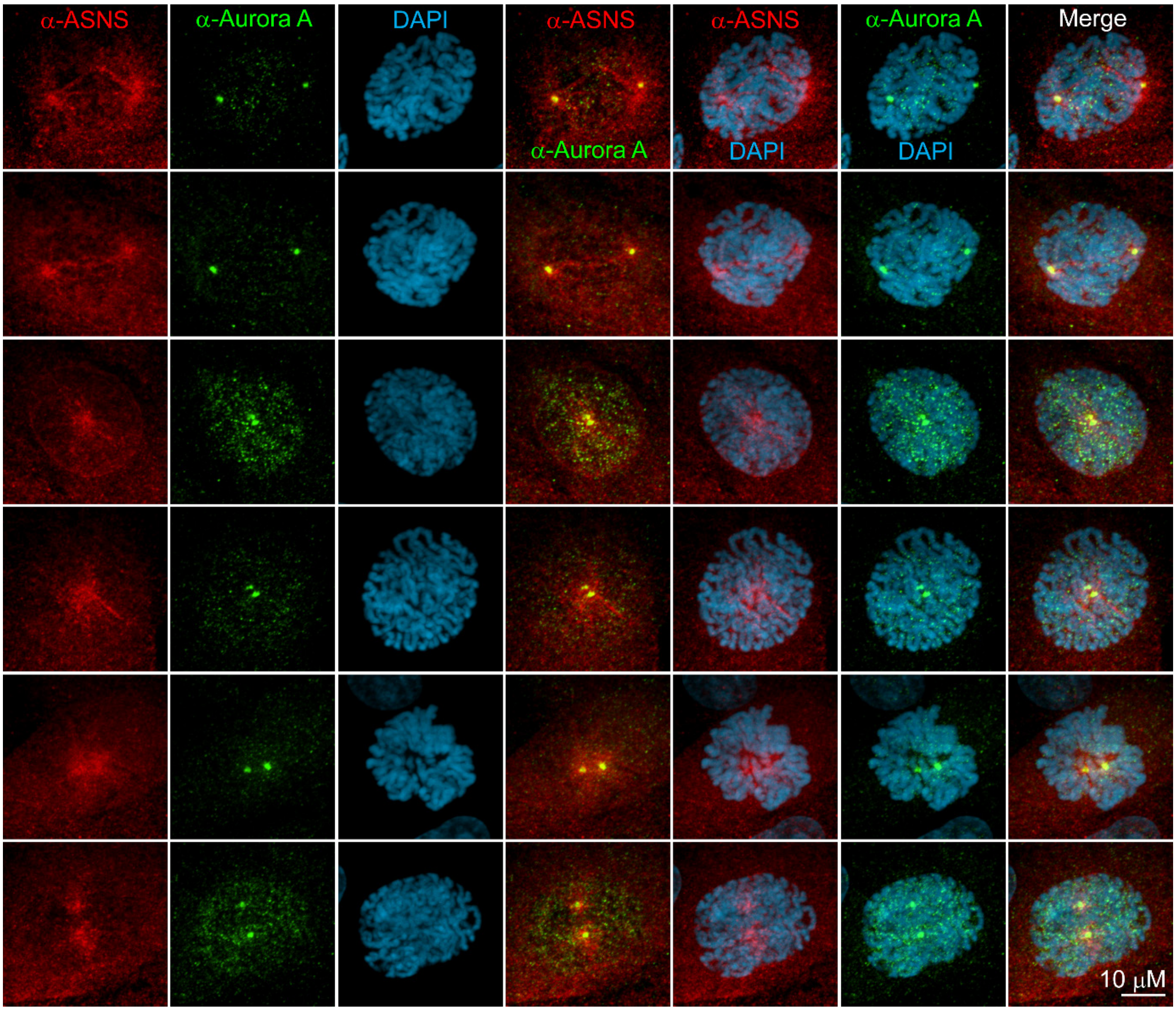

Fig. S5. Human asparagine synthetase clustered around centrosomes at the onset of mitosis. RPE-1 cells were grown for 2 days, fixed with paraformaldehyde, and immuno-stained with anti-hASNS (red) and anti-Aurora A (green).

Biology Open • Supplementary information

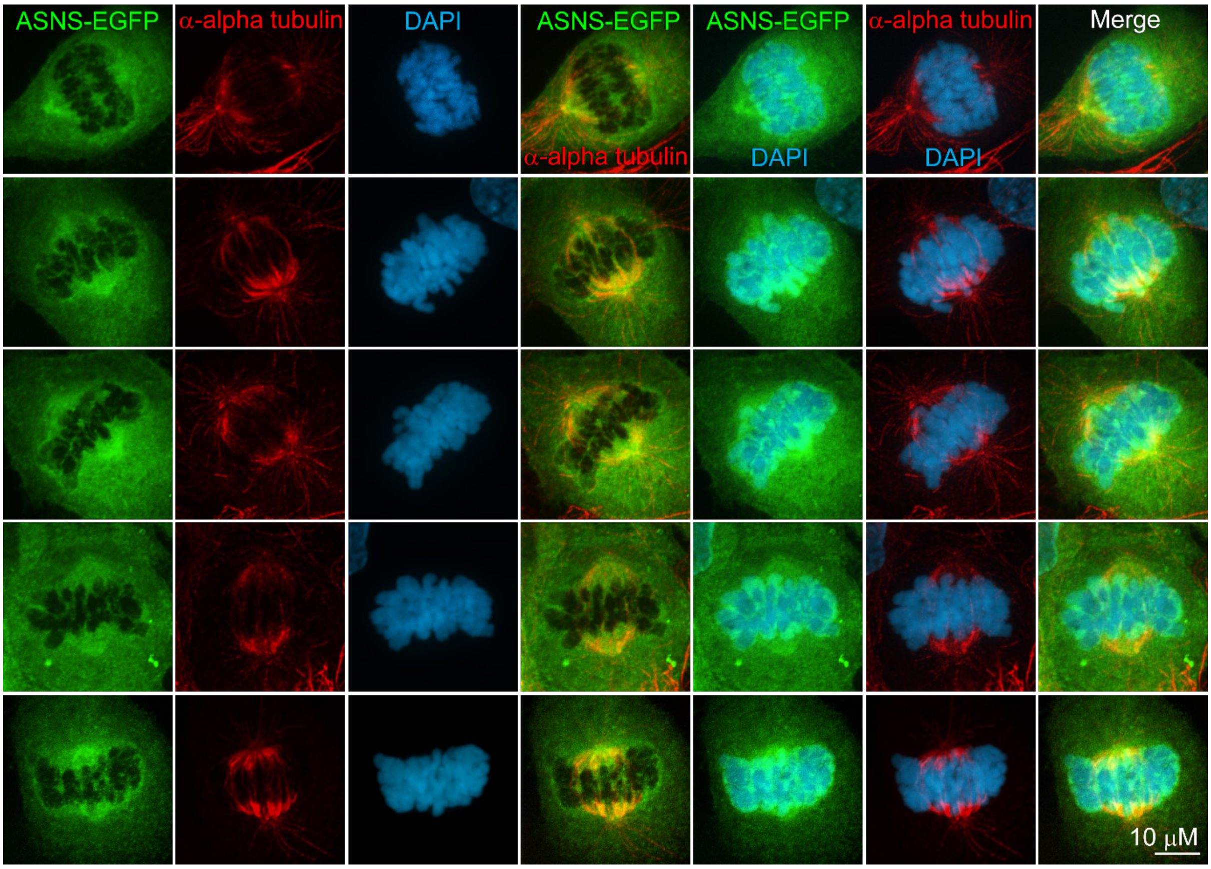

Fig. S6. ASNS-EGFP also showed mitotic spindle-like pattern. RPE-1 cells stably expressing ASNS-EGFP were grown for 3 days, fixed with paraformaldehyde, and immuno-stained with anti-alpha tubulin (red).

Biology Open • Supplementary information

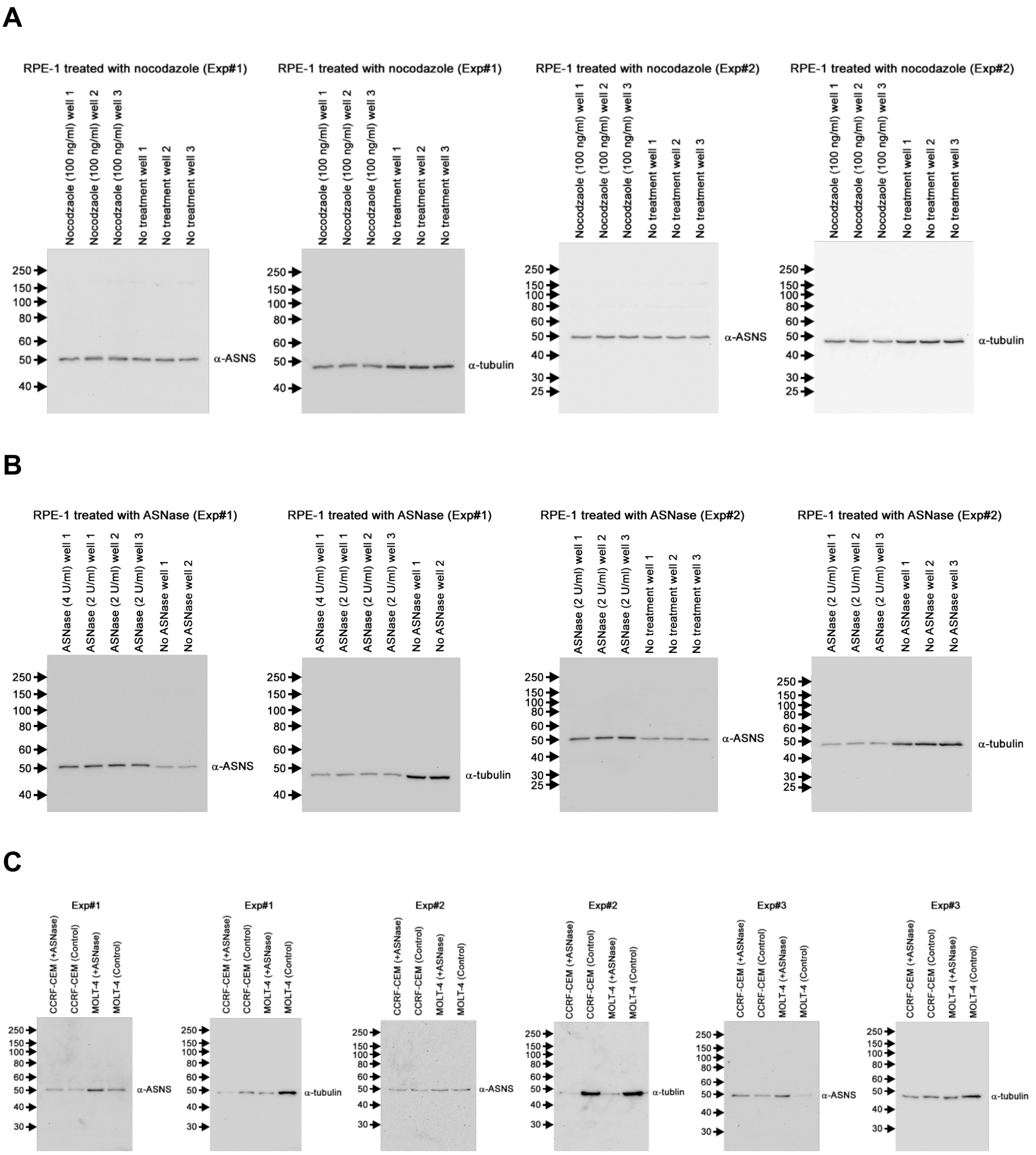

Fig. S7. Full blots of the blots shown in Fig. 3. Each experiment used the same blot for probing with anti-hASNS and anti-alpha tubulin, one at a time. Stripping buffer [62.5 mM Tris-HCl pH 6.8, 2% (w/v) SDS, 0.7% (w/v) BME] was used to remove previous antibody from the blot prior to addition of new antibody. (**A**), (**B**), and (**C**) are arranged in the same order as shown in Fig 3.
